# Differential Expression Levels of Integrin α6 Enable the Selective Identification and Isolation of Atrial and Ventricular Cardiomyocytes

**DOI:** 10.1371/journal.pone.0143538

**Published:** 2015-11-30

**Authors:** Anne Maria Wiencierz, Manuel Kernbach, Josephine Ecklebe, Gustavo Monnerat, Stefan Tomiuk, Alexandra Raulf, Peter Christalla, Daniela Malan, Michael Hesse, Andreas Bosio, Bernd K. Fleischmann, Dominik Eckardt

**Affiliations:** 1 Miltenyi Biotec GmbH, Bergisch Gladbach, Germany; 2 Institute of Physiology I, Life and Brain Center, University of Bonn, Bonn, Germany; The University of Queensland, AUSTRALIA

## Abstract

**Rationale:**

Central questions such as cardiomyocyte subtype emergence during cardiogenesis or the availability of cardiomyocyte subtypes for cell replacement therapy require selective identification and purification of atrial and ventricular cardiomyocytes. However, current methodologies do not allow for a transgene-free selective isolation of atrial or ventricular cardiomyocytes due to the lack of subtype specific cell surface markers.

**Methods and Results:**

In order to develop cell surface marker-based isolation procedures for cardiomyocyte subtypes, we performed an antibody-based screening on embryonic mouse hearts. Our data indicate that atrial and ventricular cardiomyocytes are characterized by differential expression of integrin α6 (ITGA6) throughout development and in the adult heart. We discovered that the expression level of this surface marker correlates with the intracellular subtype-specific expression of MLC-2a and MLC-2v on the single cell level and thereby enables the discrimination of cardiomyocyte subtypes by flow cytometry. Based on the differential expression of ITGA6 in atria and ventricles during cardiogenesis, we developed purification protocols for atrial and ventricular cardiomyocytes from mouse hearts. Atrial and ventricular identities of sorted cells were confirmed by expression profiling and patch clamp analysis.

**Conclusion:**

Here, we introduce a non-genetic, antibody-based approach to specifically isolate highly pure and viable atrial and ventricular cardiomyocytes from mouse hearts of various developmental stages. This will facilitate in-depth characterization of the individual cellular subsets and support translational research applications.

## Introduction

The four-chambered mammalian heart comprises different muscle cell populations, for example chamber-specific atrial and ventricular cardiomyocytes (CMs). As they differ in origin, electrophysiological properties and gene expression, they are of special interest in heart research and regenerative medicine. Due to the lack of appropriate isolation strategies, the characterization and use of CM subtypes is limited. Several purification methods have been described to enrich CMs, such as physical separation [1], fluorescent reporter or antibiotic resistance genes [2], molecular beacons [3], labeling with mitochondrial dyes [4], or metabolic selection [5]. Aside from a subtype-specific genetic modification, none of these methods facilitate the selective enrichment of atrial or ventricular CMs.

Surface marker-dependent isolation procedures are considered to be highly efficient, less time-consuming, and moreover easily translatable to therapeutic applications. Along this line, the activated leukocyte cell adhesion molecule (ALCAM) is temporarily expressed on the surface of mouse CMs between embryonic day (E) 8.25 and 10.5 [6] and has been used for the enrichment of human PSC-derived CMs [7]. Scavone and co-workers found a transient correlation between ALCAM expression and the expression of the pacemaker-specific, hyperpolarization-activated cyclic nucleotide-gated channel (HCN) 4 during mouse heart development by immunofluorescence [8]. Based on this finding, the authors were capable of isolating mesodermal progenitors from differentiating mouse pluripotent stem cells (PSCs), which were enriched in pacemaker-specific transcripts.

Applying flow cytometry analysis, Pontén and co-workers discovered that the vascular cell adhesion molecule (VCAM) 1 is a transient cell surface marker of mouse embryonic CMs and were able to purify E9.5–11.5 CMs [9]. VCAM-1 was also identified as a CM surface marker by Uosaki et al. and used for the isolation of human PSC-derived CMs [10].

Although the surface marker-dependent isolation of viable CMs using antibodies against ALCAM or VCAM-1 has been delineated, both markers have major drawbacks: first, their usage is restricted to certain stages of development and, second, both markers cannot discriminate between atrial and ventricular CMs.

So far, several intracellular proteins have been used to distinguish atrial from ventricular CMs. Atrial CMs are often specified by the presence of the myosin light chain 2a (MLC-2a, *Myl7*), the T box transcription factor 5 (*Tbx5*), the chicken ovalbumin upstream promoter transcription factor 2 (COUP-TFII, *Nr2f2*) or the connexin-40 (*Gja5*) [11] and by secretion of peptides like the fibroblast growth factor 12 (*Fgf12*) and the atrial natriuretic factor (ANF, *Nppa*) [12,13]. In contrast, ventricular CMs are characterized by the presence of the ventricle-specific MLC-2v (*Myl2*), the hairy-related transcription factor 2 (HRT-2, *Hey2*) or the Iroquois homeobox protein 4 (*Irx4*) [11,14,15].

The spatiotemporal expression of different integrin subunits in the developing mouse heart has been previously described [16], and just recently, Tarnawski and co-workers described a surface-marker combination of integrins α1 (ITGA1), α5 (ITGA5), α6 (ITGA6), and N-cadherin that could distinguish between different cardiomyocyte subtypes at E11.5 of mouse heart development [17].

Based on a surface-marker screen on E13.5 mouse hearts, we introduce ITGA6 to discriminate between atrial and ventricular CMs due to differential protein expression intensities. Using flow cytometry, we unambiguously show a direct correlation of ITGA6 with CM subtype-specific intracellular proteins in the same cell at this stage of mouse heart development.

In contrast to previously described markers, we show that differential ITGA6 expression persists throughout development up to the adult heart. Based on this, we developed isolation strategies using ITGA6 expression in combination with either ERBB-2 co-labeling or a pre-enrichment step to selectively enrich the desired CM subtype out of mixed cell populations.

## Materials and Methods

### Ethical statement

This study was conducted in accordance with the German animal protection law and with the European Communities Council Directive 2010/63/EU for the protection of animals used for experimental purposes. All experiments were approved by the North Rhine Westphalia State Agency for Nature, Environment and Consumer Protection. The permission is issued to Miltenyi Biotec GmbH (84–02.05.20.12.215).

### Dissociation of embryonic and neonatal heart tissue

The animals were maintained under specific pathogen-free conditions according to the recommendations of the Federation of European Laboratory Animal Science Association. Embryonic (CD1, wild-type, E11.5 –E17.5) or neonatal (P2) whole hearts or atrial and ventricular fractions were pooled and dissociated either manually using Collagenase B as described [18] or automated using the Neonatal Heart Dissociation Kit (Miltenyi Biotec) in combination with the gentleMACS™ Dissociator (Miltenyi Biotec). Blood cells were removed by red blood cell lysis or labeled with anti-mouse CD45 and anti-Ter119 antibodies to exclude them from the flow cytometry analysis.

### Antibody-based surface marker screen

Mouse hearts were isolated from embryos (CD1, wild-type, E13.5). The atria were mechanically removed from the ventricles. Both tissues fractions were independently dissociated and screened for surface markers with our in-house antibody library against mouse surface markers. 10^4^ atrial or 10^5^ ventricular cells were incubated with FcR Blocking Reagent (Miltenyi Biotec) and then resuspended in 100 μL of staining solution (antibody plus PBS/0.5% BSA/2 mM EDTA). Incubation for 10 min at 4°C was eventually followed by an incubation of the secondary antibody. To exclude blood cells from the analysis, each sample was co-stained with fluorochrome-coupled antibodies against murine CD45 and Ter119 (all Miltenyi Biotec). A counterstaining with PE rat anti-mouse CD166 (ALCAM) (eBioscience, 1:100) was applied as far as possible. The entire list of tested antibodies, fluorochromes, staining titers, and respective co-labeling are provided in S1 Table. Partial or full overlap with ALCAM expression indicated candidate markers for CMs (enrichment) or non-myocytes (depletion). The ratio of cells labeled with the antibody of interest served as a secondary criterion. The labeled cells were resuspended in an adequate volume of staining buffer for flow cytometry analysis. Staining and measurement were performed in 96-well U bottom plates. Samples were measured on a MACSQuant^®^ Analyzer (Miltenyi Biotec). Doublets, cell debris and dead cells (identified by propidium iodide, Sigma, 20 μg/mL) were excluded from the analysis. Analysis gates were set according to unstained or secondary antibody controls. Labeling frequencies were calculated by subtraction of the background value of the corresponding control sample. Negative values were set to 0.00.

### Flow cytometry analysis

Cells obtained from dissociated hearts were incubated with FcR Blocking Reagent (Miltenyi Biotec) and then labeled with antibodies in buffer (PBS/0.5% BSA/2 mM EDTA) for 10 min at 4°C. Sarcomeric proteins were co-labeled after fixation and permeabilization (Inside Stain Kit, Miltenyi Biotec), samples were measured on a MACSQuant^®^ Analyzer (Miltenyi Biotec). Doublets, cell debris and blood cells were excluded from the analysis. When measuring non-fixed samples, dead cells were identified by propidium iodide (PI; Sigma, 20 μg/mL) and excluded from the analysis. Analysis gates were set according to unstained, fluorescence minus one (FMO) or secondary antibody controls. For ITGA6 labeling the PE, APC, or FITC rat anti-human and mouse CD49f antibodies (clone GoH3) were used (all Miltenyi Biotec,1:11). For ITGA5 labeling the PE rat anti-mouse CD49e antibody (clone 5H10-27, BD Pharmingen, 1:50) was used. Other antibodies used: PE, APC, FITC or VioBlue^®^ rat anti-mouse CD45 (clone 30F11), rat anti-mouse Ter119 (clone Ter-119), rat anti-mouse IgG1 (clone X-56), rat anti-mouse IgG2ab (clone X-57) (all Miltenyi Biotec,1:11); PE rat anti-mouse ErbB-2/Her2 (clone 666521, R&D Systems, 1:10); mouse anti-(sarcomeric) alpha actinin (clone EA-53, isotype IgG1, unconjugated, Sigma, 1:800; FITC-conjugated, 2 μg/mL), mouse anti-MLC-2A (clone 56F5, isotype IgG2b, Synaptic systems, 1:100), mouse anti-MLC-2V (clone 330G5, isotype IgG2a, Synaptic Systems, 1:100); Alexa Fluor^®^ 488 goat anti-mouse IgG (H+L) (polyclonal, Invitrogen, 1:400).

### Flow sorting of embryonic and neonatal cardiomyocytes

Cells obtained from embryonic mouse hearts (E15.5) were labeled with the PE rat anti-mouse ErbB-2/Her2 (clone 666521, R&D Systems, 1:10) only or co-labeled with PE rat anti-mouse ErbB-2/Her2 (clone 666521, R&D Systems, 1:10) and the FITC rat anti-human and mouse CD49f (clone GoH3, Miltenyi Biotec, 1:11). Cells were resuspended in buffer and sorted on a FACSVantage SETM cell sorter (BD). Dead cells were excluded by PI staining. Sorting gates were set on PI^-^/ERBB-2^+^ cells only or on PI^-^/ERBB-2^+^/ITGA6^low^ and PI^-^/ERBB-2^+^/ITGA6^high^ cells. Neonatal CMs were purified from P2 neonatal mouse hearts using the Neonatal Cardiomyocyte Isolation Kit, mouse (Miltenyi Biotec). The enriched CM fraction was then labeled with the PE-conjugated CD49f antibody, resuspended in buffer and sorted on a FACSVantage SE^TM^ cell sorter (BD). Sorting gates were set on PI^-^/ITGA6^low^ and ITGA6^high^ cells. Flow cytometry analysis before and after cell sorting was performed on a MACSQuant^®^ Analyzer (Miltenyi Biotec). Sorted cell fractions were seeded on fibronectin-coated 24 multi-well plates at a density of 2–4 x 10^5^ cells per well and cultivated for up to 48 h (DMEM, Miltenyi Biotec; 10% FCS, PAA; 1x Penicillin/Streptomycin, PAA).

### Immunofluorescence analysis of sorted embryonic cardiomyocytes

ERBB-2^+^/ITGA6^low^ and ITGA6^high^ sorted cell cultures were fixed with 4% PFA (15 min, room temperature) and blocked with 5% goat serum (Sigma) in PBS/0.01% Triton at 4°C over night. Cells were labeled by sequential incubations with the primary antibody (45 min; mouse anti-(sarcomeric) alpha actinin antibody, Sigma, 1:800; Ki-67, BD Pharmingen, 1:100), the secondary antibody (30 min; Alexa Fluor^®^ 594 goat anti-mouse IgG (H+L), Invitrogen, 1:400) and the nuclear dye (5 min; TOTO-3 iodide, Invitrogen, 1:800; DAPI, Sigma, 1:50,000). Cells were analyzed with a Nikon Eclipse TS100 with T1-FM epi-fluorescence attachment (Nikon) or an LSM710 (Zeiss). DAPI^+^ and Ki-67^+^ nuclei were manually counted for both fractions and calculated as ratio of Ki-67/DAPI.

### Gene expression analysis of sorted embryonic and neonatal cardiomyocytes

E15.5 ERBB-2^+^/ITGA6^low^ and ERBB-2^+^/ITGA6^high^ cells as well as P2 ITGA6^low^ and ITGA6^high^ CMs were sorted from mouse hearts in four independent experiments each and subjected to gene expression analysis using Agilent Whole Mouse Genome Oligo Microarrays (8x60K, Design ID 028005) as previously described [19,20]. In brief, total RNA was isolated using the NucleoSpin RNA Kit (Macherey-Nagel) and amplified and labeled with the Low Input Quick Amp Labeling Kit (one-color, Agilent Technologies). Cy3-labeled fragmented cRNA was hybridized over night (17 hours, 65°C) to the microarrays. The obtained data were quantile normalized and transformed to logarithms to the base of 2. Hierarchical clustering was conducted using the MultiExperimentViewer 4.9.0. Differentially expressed genes were determined by a combination of statistical tests (ANOVA, Benjamini-Hochberg correction for multiple testing p ≤ 0.05, Tukey post-hoc test p ≤ 0.05) and effect size (3x). The mRNA data discussed in this publication has been deposited in NCBI's Gene Expression Omnibus and are accessible through GEO Series accession number GSE57131 (http://www.ncbi.nlm.nih.gov/geo/query/acc.cgi?acc=GSE57131).

### Whole-cell patch clamp analysis of sorted embryonic cardiomyocyte subtypes

E15.5 hearts were flow sorted using a BD Influx or BD Aria III, re-plated and cultivated for two days (IMDM w/ 20% FCS, 0.1 mM non essential amino acids, 50 μg/ml penicillin and streptomycin, 0.1 mM β-mercaptoethanol). Action potentials from spontaneously beating CMs were recorded using the patch clamp technique in the current clamp configuration, as reported previously [21]. All experiments were performed at 37°C. Cells were tested for voltage activated channels by running 250 ms lasting depolarizing ramps from -120 mV to 50 mV (holding potential: -50 mV) in the voltage-clamp mode. Statistical analysis was performed using unpaired t-tests with Graph Pad Prism 5 and LabChart 7 (AD Instruments) software for data analysis. Cells were analyzed from three independent flow sorting experiments and the results are expressed as mean ± standard error of the mean (SEM); a value of p ≤ 0.05 was considered significant.

## Results

### Antibody-based surface marker screen on embryonic mouse heart

We considered embryonic mouse hearts of E13.5 as a good model system to study developing CM subtypes because the four-chambered heart is established at this developmental stage while the CMs are still immature. In order to identify CM subtype-specific surface markers, we mechanically removed atria from ventricles. Both tissue fractions were independently dissociated and analyzed (S1A and S1B Fig). Using this model system we carried out a surface marker screen with 170 anti-mouse antibodies on both tissue fractions. The entire list of tested antibodies, staining conditions and frequencies are provided in S1 Table.

**Fig 1:**
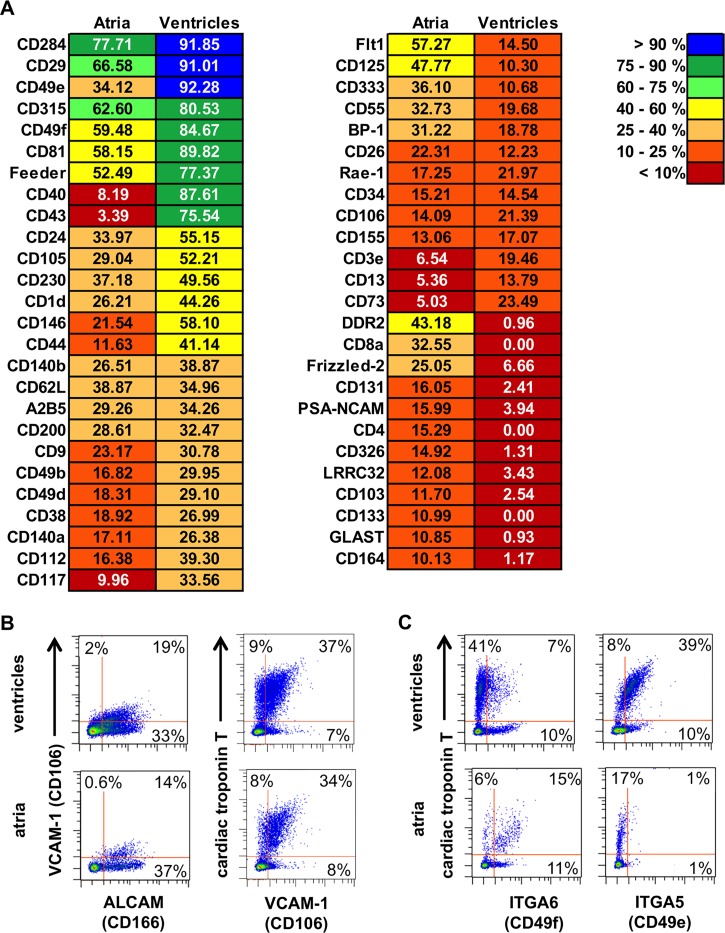
Antibody-based surface marker screening of mechanically separated atrial and ventricular fraction (E13.5). **(A)** List of all markers that were expressed by a minimum of 10% of the cells from either atrial or ventricular cells. The expression frequency is color-coded. **(B)** Density plots, left panel, co-labeling of ALCAM and VCAM-1 on both fractions as resulted from the antibody screen. Right panel, validation of VCAM-1 as CM marker by co-labeling of VCAM-1 with cardiac troponin T. **(C)** Density plots, validation of differential expression of ITGA6 (left panel) and ITGA5 (right panel) by co-labeling with an antibody against cardiac troponin T.

From the candidates we first selected antibodies that labeled more than 10% of non-blood cells in at least one of the cardiac chamber fractions resulting in a list of 51 antibodies (Fig 1A). Next we chose antibodies either detecting co-expression with ALCAM, previously described as CM marker [6, 7, 8], or marking a strong difference of antigen expression between atria and ventricles. Validation experiments analyzed potential co-expression of cardiac troponin T and the respective surface marker candidates.

As expected, we observed a full overlap of ALCAM and VCAM-1, previously described as transient surface marker of embryonic CMs [9]. We could show that at this stage of development 80% of the CMs in both fractions co-express VCAM-1 (Fig 1B). Apart from VCAM-1 we noticed additional candidates associated with endothelium. The liver sinusoidal endothelial cell marker LSEC (CD146) was found on E13.5 CMs in both fractions, but its expression was neither restricted to CMs nor differentially expressed in the atrial and ventricular fraction (S1C Fig). The endothelial cell marker Endoglin (CD105) although expressed in embryonic heart was absent from CMs at this stage (S1C Fig). Therefore, both markers were not further investigated.

We also noticed expression of several integrin chains among the 51 markers. Whereas ITGA2 (CD49b) was not expressed on embryonic CMs (S1C Fig), two integrin α chains, α5 (ITGA5, CD49e) and α6 (ITGA6, CD49f), were strongly expressed on and highly differed between the atrial and ventricular fraction. Validation experiments revealed a bright ITGA6 staining on CMs in the atrial cell fraction (Fig 1C, left panel). In contrast to this, co-labeling with the antibody against ITGA5 resulted in a distinct staining of the ventricular CMs (Fig 1C, right panel). Therefore, we focused on ITGA5 and ITGA6 to further investigate their differential cardiac expression pattern.

### ITGA5 and ITGA6 are differentially expressed on atrial and ventricular cardiomyocytes

**Fig 2:**
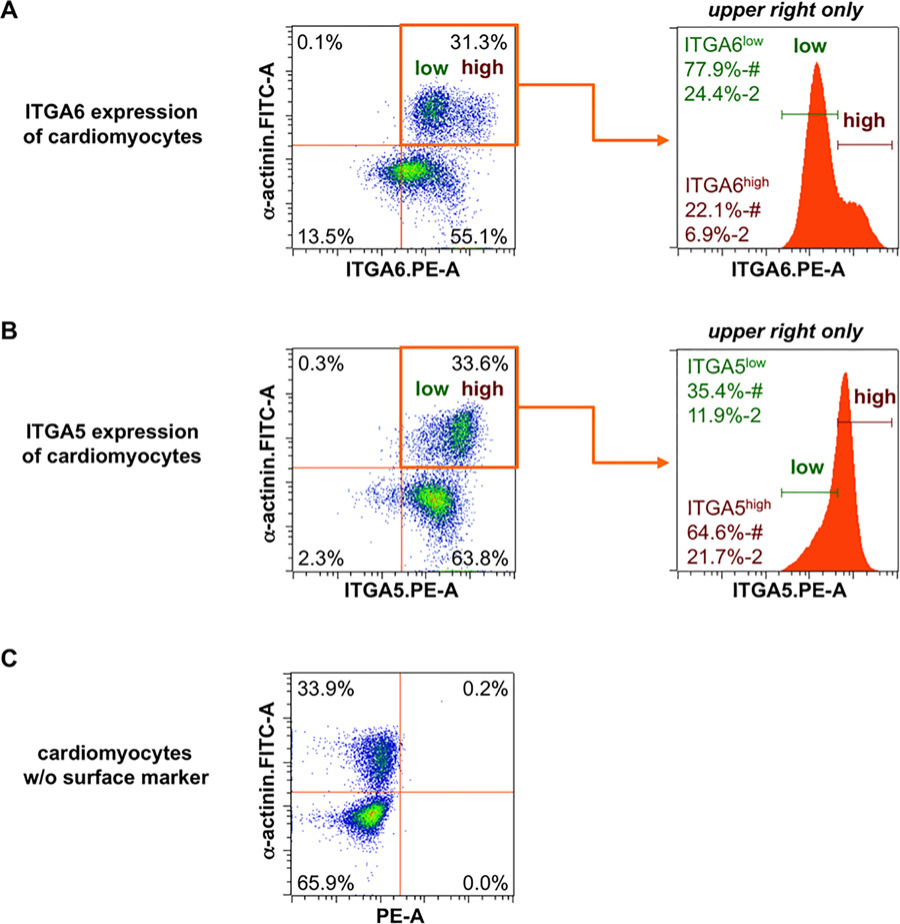
Embryonic cardiomyocytes co-express ITGA5 and ITGA6. E13.5 mouse hearts were manually dissociated and co-labeled with antibodies against ITGA6 and α-actinin **(A)** or ITGA5 and α-actinin **(B)**. Histograms display surface marker expression of the upper right quadrant only. Surface marker segregate low (green) and high (red) expressing CMs. **(C)** FMO control, intracellular labeling of CMs without surface marker staining. “%” and “%-#” refer to the current gate, “%-2” refers to the parent gate.

After the initial screening and validation experiments on chamber fractions, we next analyzed expression of ITGA5 and ITGA6 in whole heart preparations. As exemplary shown in Fig 2 virtually all E13.5 CMs expressed ITGA6 (Fig 2A) and ITGA5 (Fig 2B). ITGA6-expressing CMs segregated into a subpopulation with low fluorescence intensity (ITGA6^low^) and one with high fluorescence intensity (ITGA6^high^) (Fig 2A): 77.9% of CMs (24.4% of heart cells) were ITGA6^low^ and 22.1% of CMs (6.9% of heart cells) were ITGA6^high^. Similar to ITGA6, ITGA5-positive CMs could be subdivided into two populations based on their fluorescence intensity. As depicted in Fig 2B, 64.6% of CMs (21.7% of heart cells) were ITGA5^high^ and 35.4% of the CMs (11.9% of heart cells) were ITGA5^low^.

**Fig 3:**
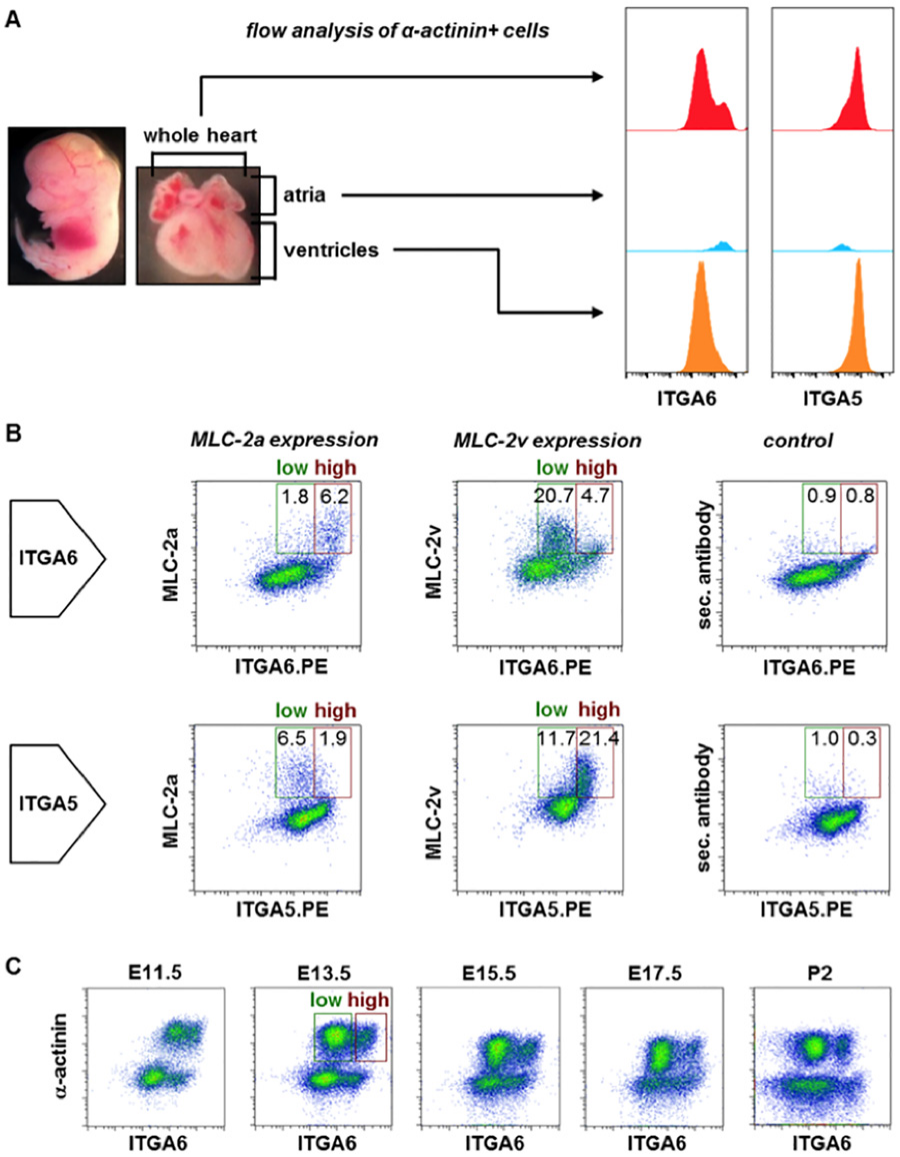
Differential expression of ITGA5 and ITGA6 on atrial and ventricular cardiomyocytes. **(A)** E13.5 whole hearts and mechanically separated atrial and ventricular tissue were co-labeled for ITGA6 or ITGA5 and α-actinin. Histograms, ITGA6 or ITGA5 expression gated on α-actinin+ cells. **(B)** E13.5 whole-heart preparations co-stained with antibodies against ITGA6 or ITGA5 and MLC-2a or MLC-2v (labeled with AlexaFluor^®^ 488 goat anti-mouse IgG). Analysis gates set according to the secondary antibody control. Rectangles indicate ITGA6 low (green) and high (red) expressing myocytes. **(C)** Co-labeling of E11.5 –P2 mouse hearts for ITGA6 and α-actinin.

When comparing the ITGA6 expression pattern in whole-hearts with atrial and ventricular preparations we detected two peaks for CMs in whole-heart preparations, but only one peak of high fluorescence intensity in the atrial and one of low intensity in the ventricular tissue fraction (Fig 3A). Regarding ITGA5 expression we found one tailing peak in whole-heart preparations, which could be split into one at low intensity for the atrial and one at high intensity for the ventricular preparation.

Next we co-labeled ITGA5 or ITGA6 with chamber-specific MLC-2a and MLC-2v antibodies (Fig 3B). Gating boundaries of MLC-2a and -2v staining were set according to the secondary antibody control (S2A Fig). MLC-2a^+^ cells co-expressed ITGA6^high^ (6.2%) and ITGA5^low^ (6.5%), whereas MLC-2v expression correlated with ITGA5^high^ (21.4%) and ITGA6^low^ (20.7%). This corresponded to our previous finding, and we concluded that antibodies against both surface markers could be used to separate atrial and ventricular embryonic CMs based on fluorescence intensity after respective antibody labeling. We focused on ITGA6 and investigated its expression at various stages of cardiogenesis (Fig 3C, S2B Fig). Antibody labeling revealed a differential ITGA6 fluorescence intensity of atrial and ventricular CMs as early as E11.5, which increased with age up to P2.

In addition, we tested atrial and ventricular fractions from P8 as well as from adult mouse hearts in order to evaluate whether differences in fluorescence intensity between atrial and ventricular CMs were restricted to the developing or conserved in adult heart. As shown in S2C Fig we detected only one broad peak for the CMs in the whole-heart preparations of P8 mice. But, as depicted in the histogram overlay of atrial and ventricular ITGA6 expression, there still was a higher fluorescence intensity of atrial CMs than of ventricular CMs (S2C Fig). The same applied to CMs isolated from adult atria and ventricles (S2D Fig). However, the difference in fluorescence intensity was lower than at the P2 stage.

As flow cytometry analysis indicated that ITGA6 was not restricted to CMs, but could be found on other heart cells, we sought to either combine the subtype-specific marker with a surface marker labeling all CMs or to apply a depletion strategy to remove all cardiac non-myocytes.

### Selective enrichment of embryonic and neonatal cardiomyocyte subtypes

**Fig 4:**
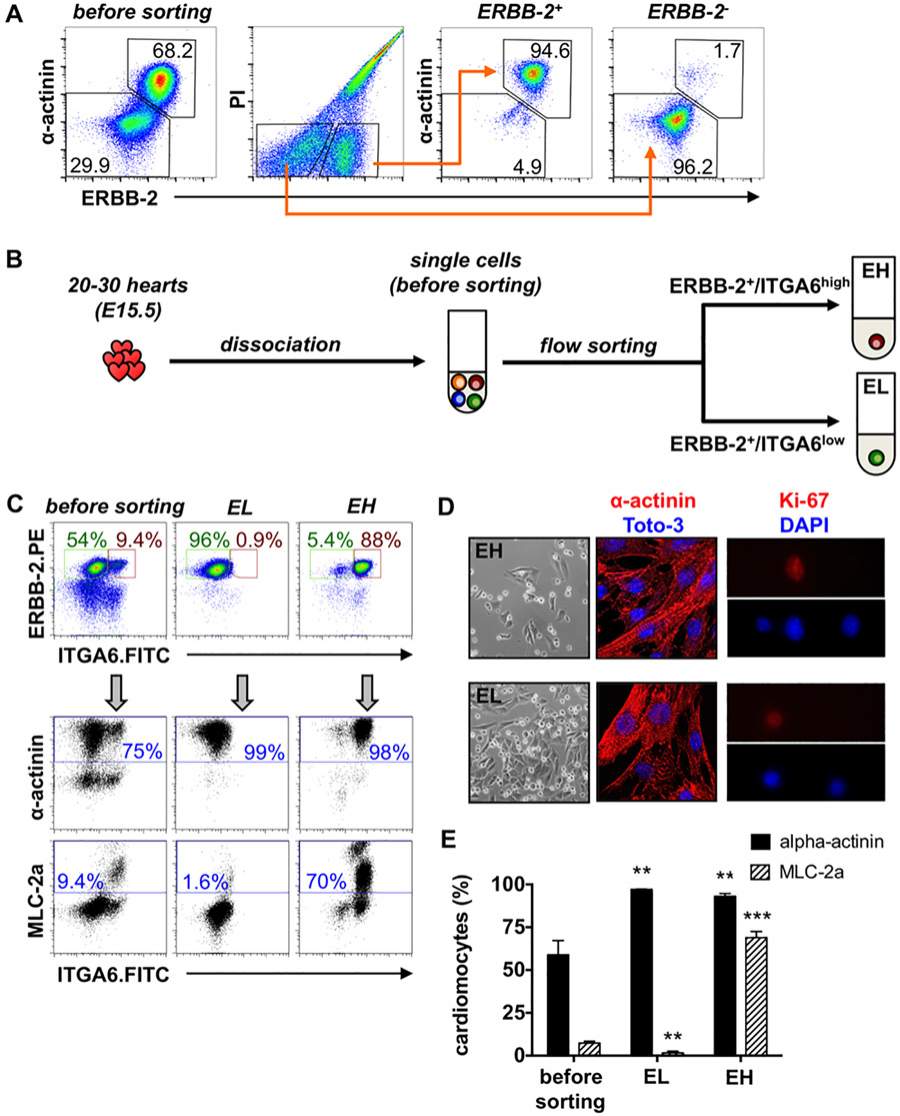
Selective enrichment of embryonic atrial and ventricular cardiomyocytes. **(A)** E15.5 CMs were purified by sorting of whole-heart suspensions into PI^-^/ERBB-2^+^ and PI^-^/ERBB-2^-^ cells. **(B)** E15.5 mouse hearts were dissociated and sorted into ERBB-2^+^/ITGA6^low^ (EL) and ERBB-2^+^/ITGA6^high^ (EH) cells. **(C)** Density plots, distribution of the cells before and after sorting. Dot plots, co-staining of the fractions with pure antibodies against α-actinin and MLC-2a (labeled with APC rat anti-mouse IgG1 or IgG2ab). **(D)** Sorted fractions were plated on fibronectin-coated dishes. Cells were attached after one day; most of them flattened but some still round. Immunofluorescence analysis of plated cells one day after sorting in terms of α-actinin cross-striation (mid panel) and of Ki-67 expression (right panel). **(E)** Statistical analysis of the CM subtype isolation with regard to the content of α-actinin and MLC-2a before and after sorting. Data are expressed as mean ± SD, n = 4. *t*-test for paired samples with** p ≤ 0.01, *** p ≤ 0.001 vs. before sorting.

We analyzed the expression of known CM surface markers such as ALCAM and VCAM-1 [6,9] and included ERBB-2 into the analysis due to its important role in CMs and heart development [22]. In full agreement with earlier reports, single cell analysis of mouse hearts of various developmental stages confirmed highly regulated expression of ALCAM and VCAM-1 with restriction to CMs at E11.5. Moreover, we found CM-restricted expression of ERBB-2 at E15.5 (S3 Fig, S1 Methods). In fact, ERBB-2 flow sorting of E15.5 hearts resulted in a potent enrichment of CMs in the ERBB-2^+^ fraction (94.6% ERBB-2^+^/α-actinin^+^); only 1.7% CM were retained in the ERBB-2^-^ fraction (Fig 4A). We therefore combined ERBB-2 with ITGA6 and sorted embryonic hearts into ERBB-2^+^/ITGA6^low^ (EL) and ERBB-2^+^/ITGA6^high^ cells (EH) (Fig 4B). As depicted in Fig 4C, this led to a high enrichment of CMs in both populations: the α-actinin frequency increased from 75% in the unsorted whole-heart preparation to 99% and 98% in EL and EH, respectively. As indicated by MLC-2a labeling, EL and EH highly differed in their composition. From initial 9.4% in the whole heart MLC-2a frequency was elevated up to 70% in the EH fraction and reduced down to 1.6% in the EL population.

The sorted cells were viable, could be cultivated and exhibited spontaneous contractions 24 h after plating (Fig 4D, S1 and S2 Videos). CM identity was confirmed by the typical cross-striated α-actinin staining pattern in all cells of both fractions (Fig 4D). Cell cycle activity of the sorted cells was analyzed by Ki-67 staining: 28 out of 205 nuclei expressed Ki-67 in the EL fraction (= 13.7%) and 42 out of 238 in the EH fraction (= 17.7%). Walsh and co-workers [23] determined a Ki-67 labeling index of ~12% for E14.5 mouse hearts which confirms our findings.

**Fig 5:**
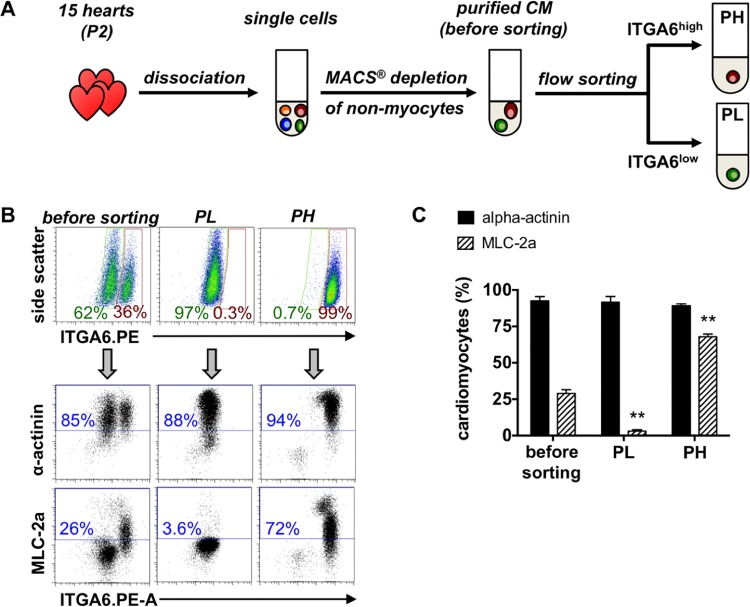
Selective enrichment of neonatal atrial and ventricular cardiomyocytes. **(A)** P2 hearts were dissociated, depleted from cardiac non-myocytes and then sorted into ITGA6^low^ (PL) and ITGA6^high^ (PH) cells. **(B)** Density plots, distribution of cells before and after sorting. Dot plots, staining of the fractions for α-actinin and MLC-2a (labeled with APC anti-mouse IgG1 or IgG2ab). **(C)** Statistical analysis of α-actinin and MLC-2a before and after sorting. Data are expressed as mean ± SD, n = 3. *t*-test for paired samples with ** p ≤ 0.01 vs. before sorting.

As ALCAM, VCAM-1 as well as ERBB-2 expression decreased after birth (S3 Fig), the two-marker sorting strategy was restricted to embryonic hearts. Therefore, neonatal CMs were first purified using magnetic cell sorting (Fig 5A), and second, were separated into CM subtypes based on ITGA6 expression intensities, i.e. ITGA6^low^ (PL) and ITGA6^high^ cells (PH). As expected from the previous experiments, the two fractions highly differed with regard to MLC-2a expression: 70% MLC-2a^+^ cells in PH, 4% MLC-2a^+^ in PL (Fig 4B). Altogether, both isolation strategies repeatedly resulted in a highly selective enrichment or depletion of MLC-2a^+^ cells from mouse hearts (Figs 4E and 5C).

### Embryonic and neonatal ITGA6^high^ and ITGA6^low^-sorted cardiomyocytes display atrial and ventricular gene expression patterns, respectively

**Fig 6:**
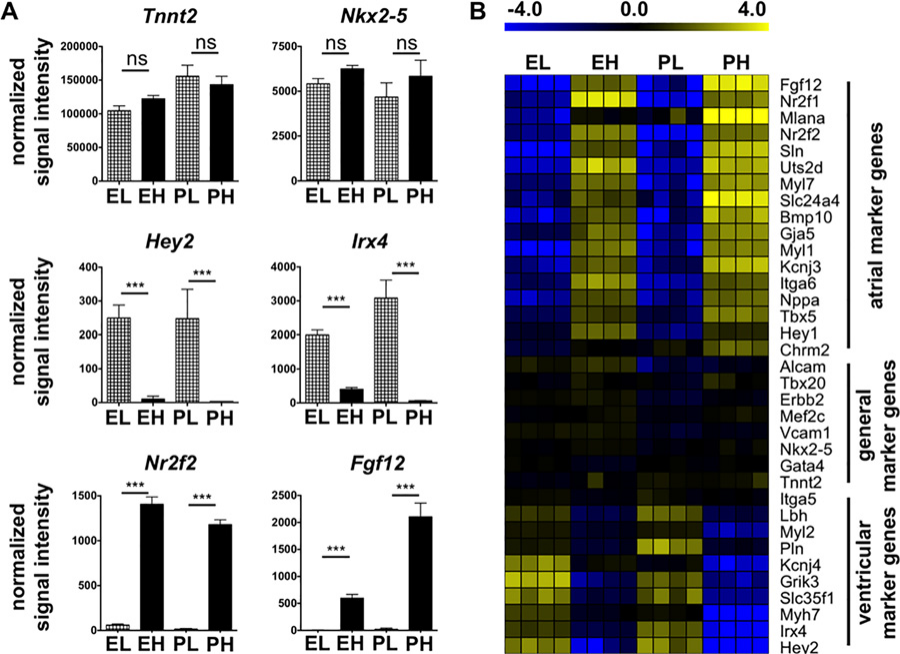
Gene expression analysis of sorted cells confirms selective enrichment of atrial and ventricular cardiomyocytes. **(A)** Normalized signal intensities of CM-specific marker genes: general CM-specific *Tnnt2* and *Nkx2-5*, ventricle-specific *Hey2* and *Irx4*, atrium-specific *Nr2f2* and *Fgf12*. Data are expressed as mean ± SD, n = 4. Statistical analysis: ANOVA, Benjamini-Hochberg correction for multiple testing p ≤ 0.05, Tukey post-hoc test *** p ≤ 0.001, ns = not significant. **(B)** Heat-map shows median-centered log2-transformed signal intensities of selected genes. The color code indicates expression relative to the gene-wise median of all samples. Abbreviation: EL = E15.5 ERBB-2^+^/ITGA6^low^, EH = E15.5 ERBB-2^+^/ITGA6^high^, PL = P2 ITGA6^low^, PH = P2 ITGA6^high^.

To further characterize the subpopulations, cells were sorted from E15.5 and P2 mouse hearts in quadruplicates and subjected to microarray analysis. A correlation analysis of the complete dataset resulted in a clear separation of the different sample groups (S4 Fig). The normalized signal intensities for general CM markers such as cardiac troponin T (*Tnnt2*) and NK2 homeobox 5 (*Nkx2-5*) were high and did not significantly vary between ITGA6^low^ (EL, PL) and ITGA6^high^ cells (EH, PH) (Fig 6A). However, several genes were restricted to one of the sorted fractions. For example, ventricle-specific transcripts *Hey2* or *Irx4* could be detected in EL and PL, but not or only at very low levels in EH and PH cells. In contrast, several genes associated with atrial myocytes were found to be weakly or not at all expressed in the EL and PL fractions, but were highly expressed in the EH and PH fractions, among them *Fgf12* and *Nr2f2* (encoding COUP-TFII).

Differentially expressed genes were determined by a combination of statistical tests and effect size (3-fold). 329 genes were higher expressed in EH and PH compared to EL and PL, 129 genes were lower expressed (S2 and S3 Tables). A list of selected genes is provided in heat-map format (Fig 6B) and in Table 1. As expected, atrial genes (e.g. *Sln*, *Gja5*, *Nppa*, *Tbx5*) were overrepresented in EH and PH, whereas ventricle-specific genes (e.g. *Lbh*, *Myh7*) were underrepresented. Corresponding to the flow cytometry data, we found an enrichment of the gene encoding MLC-2a, *Myl7*, as well as of *Itga6* in EH and PH. No difference was observed for genes encoding general CM surface markers such as *Alcam*, *Vcam1* and *Erbb2* as well as for *Itga5*. Altogether, expression analysis confirmed selective enrichment of atrial and ventricular genes in the sorted fractions.

**Table 1:** List of fold-change values of selected genes with general or subtype-specific expression in mouse cardiomyocytes.

Gene	Name	Ref.	E15.5	P2
**genes that are higher expressed in atrial CM**				
*Fgf12*	Fibroblast growth factor 12	^[^ ^13^ ^]^	119.2	122.9
*Nr2f1*	Nuclear receptor subfamily 2, group F, member 1; COUP-TFI	^[^ ^24^ ^]^	65.4	100.2
*Nr2f2*	Nuclear receptor subfamily 2, group F, member 2; COUP-TFII	^[^ ^11^ ^,^ ^24^ ^,^ ^25^ ^]^	23.7	76.6
*Sln*	Sarcolipin	^[^ ^11^ ^,^ ^24^ ^]^	74.3	72.3
*Myl7*	Myosin light chain 2a; MLC-2a	^[^ ^12^ ^,^ ^25^ ^]^	15.0	60.5
*Slc24a4*	Na/K/Ca exchanger 4	^[^ ^11^ ^,^ ^25^ ^]^	11.0	54.5
*Bmp10*	Bone morphogenetic protein 10	^[^ ^26^ ^]^	29.5	51.0
*Gja5*	Connexin-40	^[^ ^11^ ^]^	9.7	42.4
*Myl1*	Myosin light chain alkali 1/2	^[^ ^25^ ^]^	75.5	38.0
*Kcnj3*	G protein-activated inward rectifier K channel 1; Kir3.1	^[^ ^11^ ^,^ ^25^ ^]^	18.0	32.5
*Itga6*	Integrin alpha-6; ITGA6; CD49f	new	19.1	22.8
*Nppa*	Natriuretic peptide precursor A; ANF	^[^ ^11^ ^,^ ^25^ ^]^	20.3	19.5
*Tbx5*	T-box transcription factor 5	^[^ ^11^ ^,^ ^25^ ^]^	6.6	11.6
*Hey1*	Hairy-related transcription factor 1; HRT-1	^[^ ^14^ ^]^	6.2	7.1
**genes that are expressed in CM**				
*Alcam*	Activated leukocyte cell adhesion molecule; ALCAM	^[^ ^6^ ^]^	1.4	1.8
*Tbx20*	T-box transcription factor 20	^[^ ^11^ ^]^	1.3	1.7
*Erbb2*	Receptor tyrosine-protein kinase ERBB-2; Her2; Neu; CD340	^[^ ^22^ ^]^	1.2	1.6
*Vcam1*	Vascular cell adhesion protein 1; VCAM-1	^[^ ^9^ ^]^	1.0	1.4
*Nkx2-5*	NK2 homeobox 5	^[^ ^11^ ^]^	1.2	1.2
*Gata4*	GATA-binding factor 4	^[^ ^11^ ^]^	-1.5	-1.0
**genes that are higher expressed in ventricular CM**				
*Lbh*	Limb bud and heart-expressed protein	^[^ ^11^ ^,^ ^24^ ^,^ ^25^ ^]^	-5.1	-7.9
*Myl2*	Myosin light chain 2v; MLC-2v	^[^ ^25^ ^]^	-2.2	-9.0
*Pln*	Cardiac phospholamban	^[^ ^11^ ^]^	-2.6	-9.2
*Myh7*	Myosin heavy chain, beta isoform	^[^ ^25^ ^]^	-3.1	-22.3
*Irx4*	Iroquois homeobox protein 4	^[^ ^11^ ^,^ ^15^ ^,^ ^25^ ^]^	-5.0	-47.5
*Hey2*	Hairy-related transcription factor 2; HRT-2	^[^ ^11^ ^,^ ^14^ ^]^	-30.8	-67.1

Positive fold-change values indicate a higher abundance in ITGA6^high^ as compared to ITGA6^low^-sorted cells, negative values demonstrate a higher abundance in ITGA6^low^-sorted cells in comparison to ITGA6^high^. Differential gene expression was assumed for fold-change values ≥ 3.0 or ≤ -3.0.

### Embryonic ITGA6^high^ and ITGA6^low^-sorted cardiomyocytes display atrial and ventricular-like action potentials, respectively

**Fig 7:**
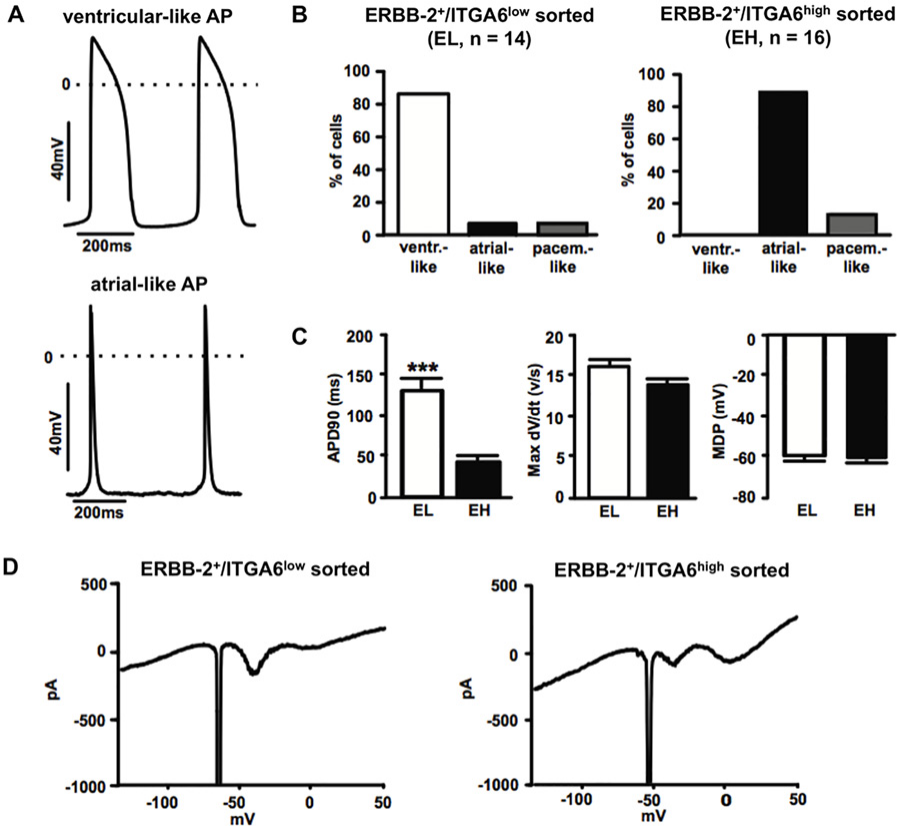
Functional subtype characterization of sorted cells confirms selective enrichment of atrial and ventricular cardiomyocytes. **(A)** Top graph, typical ventricular-like action potential (AP) of a cell from the EL group. Bottom graph, typical atrial-like AP from a CMs of the EH group. **(B)** Distribution of the cells in the two sorted groups. **(C)** Statistical analysis of AP parameters: left, action potential duration at 90% of repolarization (ADP90); mid, maximum rate of rise of the AP (max dV/dt); right, maximum diastolic polarization (MDP). Data are expressed as mean ± SEM. *** p ≤ 0.001 EL vs. EH. **(D)** Representative voltage ramps recordings from an E15.5 ERBB-2^+^/ITGA6^low^ CM (left) and an E15.5 ERBB-2^+^/ITGA6^high^ CM (right) show functional expression of inward and outward current components. Abbreviation: EL = E15.5 ERBB-2^+^/ITGA6^low^, EH = E15.5 ERBB-2^+^/ITGA6^high^, PL = P2 ITGA6^low^, PH = P2 ITGA6^high^.

To determine the viability and the functional subtype of the different CM fractions, action potentials (AP) were recorded from sorted E15.5 cells by patch-clamp experiments. 85.7% of the CMs (total n = 14) sorted with ERBB-2^+^/ITGA6^low^ (EL) exhibited typical AP shape and long AP duration characteristic for ventricular-like cells; in this group only one cell had an atrial-like and one a pacemaker-like phenotype (Fig 7A and 7B). The CM population sorted with ERBB-2^+^/ITGA6^high^ (EH) demonstrated a prevalently atrial-like phenotype with a very short AP duration (87.5%, total n = 16 cells); only two cells displayed a pacemaker-like and none a ventricular-like AP (Fig 7A and 7B). The most important AP parameters for both populations were quantified (Fig 7C) and yielded the following results: APD90: 132.6 ± 11.8 ms (EL), 47.4 ± 4.5 ms (EH); MDP: -60.1 ± 2.1 mV (EL), -61.2 ± 1.8 mV (EH); Max dV/dt: 16.3 ± 0.8 V/s (EL), 14.0 ± 0.7 V/s (EH). The two populations differed significantly for APD90. These values correspond to previous studies of murine embryonic CMs [9,27].

We also analyzed the functional expression of voltage dependent currents by applying voltage ramps. These experiments illustrated that all major components of voltage activated currents, namely Na^+^, Ca^2+^ -and inwardly- and outwardly rectifying currents are expressed in EL and EH CMs (Fig 7D). These data demonstrate the physiological viability of the sorted cells as well as the successful separation into atrial- and ventricular-like CMs [9,28].

## Discussion

To date, several methods have been described to isolate CMs, e.g. by the surface markers VCAM-1 [9,10] or ALCAM [6,7]. The first surface marker-dependent isolation of a CM subpopulation, namely pacemakers, has been described based on transient ALCAM expression [8]. The isolation of atrial and ventricular CM populations at E11.5 using integrin expression has just recently been reported [17]. Herein we present a novel surface marker-dependent purification of atrial and ventricular CMs from embryonic as well as neonatal mouse hearts.

Our antibody-based surface marker screen on embryonic mouse hearts validated previously published CM surface markers. In addition, we identified ERBB-2 as a specific surface marker for CMs at day E15.5. It is well known that ERBB-2 plays an important role in early and later stages of cardiogenesis [22,29] and we show here that ERBB-2-based flow sorting even facilitates the purification of embryonic CMs with purities of > 94%.

Main α chain integrins in CMs are α1, α5, and α7, which heterodimerize with integrin β1 to form collagen, fibronectin and laminin-binding receptors, respectively. Additionally described is the expression of α6, α9 and α10 [16]. Nevertheless, integrins are found throughout the body, and subunit expression varies e.g. with developmental stages and cell-type. While ITGA5 is found mostly on embryonic and neonatal CMs, it is replaced by integrin α7 in the adult heart. Data from *in situ* hybridization and immunohistochemistry indicated expression of ITGA6 in the developing heart [30].

In our antibody screen, we discovered that atrial and ventricular CMs are characterized by differential expression of ITGA6 and ITGA5 at E13.5. This is in line with the findings of Tarnaswki and co-worker who used a triplet to integrin α chains for the isolation of CM subtypes a E11.5 [17]. Using qRT-PCR and electrophysiology they classified the flow sorted populations as atrial (ITGA6^+^ITGA1^-^ITGA5^-^), ventricular compact (ITGA6^-^ITGA1^+^ITGA5^+^), and ventricular trabecular cells (ITGA6^+^ITGA1^+^ITGA5^+^).

Our single cell analysis of mechanically separated E13.5 hearts revealed that the expression level of a single surface marker, i.e. either ITGA6 or ITGA5, can discriminate between atrial and ventricular CMs. Additionally, we could directly relate the variation in integrin expression levels to the expression of MLC-2a (ITGA6^high^, ITGA5^low^) and MLC-2v (ITGA6^low^, ITGA5^high^)

More importantly, we show that the differential ITGA6 expression in atrial and ventricular CMs is not a transient feature as it is for ITGA5 but persists throughout heart development and could even be demonstrated in adult hearts. This makes ITGA6 stand out from all previously identified CM surface markers.

Although flow cytometry-based discrimination of CM subtypes requires only one surface marker, ITGA6, CM purification needs either a second surface marker or a CM pre-enrichment step. We unequivocally demonstrate that ERBB-2 is a suitable second marker for discrimination of CMs and non-CMs at E15.5 and that magnetic pre-depletion of non-myocytes facilitates the isolation of highly pure atrial and ventricular CMs from neonatal hearts. Based on the expression kinetics we suggest VCAM-1 or ALCAM as additional CM markers for the isolation of CM subtypes of earlier embryonic stages. In fact, an efficient purification of CMs from E9.5 –E12.5 mouse hearts by flow sorting of VCAM-1^+^/PECAM-1^-^ cells has been described [9]. Combined with ITGA6 we assume this could be used as an alternative purification strategy to isolate CM subtypes at these stages as well.

Apart from flow cytometry, we could also show the segregation of atrial and ventricular CMs by microarray analysis. In particular, the genes encoding key transcription factors of atrial and ventricular identity [11] such as the orphan nuclear receptor COUPT-TFII and HEY2 were found among the most differentially regulated genes and were absent from the ITGA6^low^ and ITGA6^high^ sorted fractions, respectively. Therefore, we were wondering whether ITGA6 is linked to the transcriptional networks that render atrial and ventricular identity. Recently, it has been described that COUP-TFII determines atrial identity since it promotes atrial (*Tbx5*) gene expression by tethering to SP1 binding sites and it represses ventricular gene expression (*Hey2*, *Irx4*) [11,29]. Therefore, we analyzed the *Itga6* core promoter in terms of regulatory elements using the TRANSFAC database (Rel. 3.3 06-01-1998). Based on an 85.0 threshold, 18 high-scoring transcription factor binding sites were identified in the *Itga6* promoter, among them seven Sp1 sites (S4 Table). This could provide a first possible link of ITGA6 to the transcription factor network specifying atrial identity. Further investigations like reporter gene and ChiP assays are required to better understand the potentially functional role of ITGA6 during establishment of CM subtypes.

In summary, we established a novel method to selectively identify and isolate primary atrial and ventricular CMs from mouse heart based on distinct expression levels of ITGA6. Our newly developed method facilitates the enrichment of viable CM subtypes suitable for any downstream application like physiological or biochemical analysis and *in vitro* cultivation. Although beyond the scope of our study, differential expression levels of ITGA6 may as well be detected on PSC-derived CMs or subtypes and as well enable their identification and selective enrichment.

## Supporting Information

S1 Fig Intracellular and surface marker expression of manually separated and dissociated atria and ventricles. **(A)** Density plots show representative flow analysis of the cardiomyocyte content in both fractions as indicated by α-actinin staining. **(B)** Identity of atrial and ventricular cells was analyzed by qRT-PCR with regard to transcript levels of the ventricle-specific *Myl2*, encoding MLC-2v, the atrium-specific *Myl7* (MLC-2a) and *Nppa* (ANF) as well as the endothelium-specific *Cdh5* (VE-cadherin). Gene expression was calculated relative to *Gapdh* and analyzed using the ΔΔCT method (S1 Methods). The fold change is calculated as a relative change of atrial versus ventricular expression. Bar graph shows mean ± SD, n = 5. **(C)** Density plots, co-labeling of surface markers LSEC, Endoglin, and ITGA2 with intracellular cardiac troponin T.
(TIF)

S2 Fig Differential expression of ITGA6 in atria and ventricles at various developmental and postnatal stages. **(A)** Exemplary analysis. Determination of doublets by displaying FSC-A (X-axis) versus FSC-H (Y-axis); all events that are not on the diagonal are considered doublets and excluded from the analysis (NOT-gate, red dashed line). Definition of the cell population by displaying FSC-A versus SSC-A; only events of the main population are considered cells and included in the analysis (black line). Unless blood cells are not removed by lysis, they are labeled with antibodies against CD45 and Ter119 in one fluorescence channel (e.g. APC) and detected by displaying blood cells versus FSC-A; all CD45^+^ or Ter119^+^ cells are excluded from the analysis (NOT-gate, purple dashed line). Further analysis gates are set on unstained cells or secondary antibody controls. % refers to the entirety of the cells displayed in the plot which depend on the parent gates. **(B)** Representative flow analysis of mouse hearts of various developmental stages. Density plots, whole-heart cell suspensions were co-labeled with antibodies against ITGA6 and α-actinin. Histograms, ITGA6 expression gated on α-actinin^+^ cells of whole-heart (top row) and of mechanically separated atrial (mid row) and ventricular cells (bottom row). At all investigated stages atrial and ventricular CMs increasingly differ in ITGA6 expression intensity. **(C)** Histograms, cardiomyocytes were purified from P8 whole-heart, atria, and ventricles, and labeled with an antibody against ITGA6. There was only one peak detectable in the whole-heart preparation (left plot) but staining of isolated fractions revealed a remaining difference in ITGA6 expression intensity between the two fractions (right plot). Overlay: red peak = ventricular fraction, blue peak = atrial fraction. **(D)** Histogram, cardiomyocytes were isolated from adult atria and ventricles (S1 Methods), and were co-labeled with antibodies against ITGA6 and cardiac troponin T. There is a difference in ITGA6 expression intensity. Overlay: red peak = ventricular fraction, blue peak = atrial fraction. Abbreviations: A–area, H–height, FSC–forward scatter, SSC–side scatter, APC–allophycocyanin, PE–R-phycoerythrin, FITC–fluorescein.
(TIF)

S3 Fig Temporal expression pattern of ALCAM, VCAM-1 and ERBB-2 in the developing mouse heart. Representative flow analysis of mouse hearts of different developmental stages (E11.5 –P2). Density plots, whole-heart cell suspensions were co-labeled with antibodies against α-actinin (X-axis) and the surface markers ALCAM, VCAM-1 or ERBB-2 (Y-axis). Gates were set to corresponding unstained controls. As illustrated, cardiac expression of the markers was highly regulated during development. Cardiomyocyte-specific expression of ALCAM and VCAM-1 at E11.5 as well as of ERBB-2 at E15.5 is indicated by the red rectangle.
(TIF)

S4 Fig Correlation matrix showing the relationship of gene expression profiles. In four independent experiments embryonic and neonatal mouse heart cells were flow sorted and collected for gene expression analysis. The matrix was generated by unsupervised hierarchical clustering of pair-wise correlation coefficients (Pearson). Correlation coefficients are indicated by their color from 0.94 (black) to 1.0 (yellow). As depicted in the heat-map, hierarchical clustering of the complete dataset by experiments resulted in a clear separation of the different sample groups. EL = E15.5 ERBB-2^+^/ITGA6^low^, EH = E15.5 ERBB-2^+^/ITGA6^high^, PL = purified P2 CM ITGA6^low^, PH = purified P2 CM ITGA6^high^; n = 4.
(TIF)

S1 Methods Supplemental methods and materials. (DOC)

S1 Table List of tested antibodies, staining conditions and frequencies of the Antibody-based surface marker screening. (XLS)

S2 Table List of differentially expressed genes with a higher abundance in embryonic and neonatal ITGA6^high^ sorted cells (alphabetical order). Positive fold change values indicate a higher abundance in ITGA6^high^ as compared to ITGA6^low^-sorted cells; differential gene expression was defined as a fold change value ≥ 3.0.
(XLSX)

S3 Table List of differentially expressed genes with a higher abundance in embryonic and neonatal ITGA6^low^ sorted cells (alphabetical order). Negative fold change values indicate a higher abundance in ITGA6^low^ as compared to ITGA6^high^-sorted cells; differential gene expression was defined as a fold change value ≤ -3.0.
(XLSX)

S4 Table List of transcription factor binding sites in the Itga6 core promoter. (XLSX)

S1 Video Contracting flow sorted ERBB-2^+^/ITGA6^low^ cells. E15.5 mouse hearts were flow sorted into ERBB-2^+^/ITGA6^low^ cells (EL) and plated on fibronectin-coated dishes. 10 s videos were recorded from spontaneously beating cells 24 h after plating.
(AVI)

S2 Video Contracting flow sorted ERBB-2^+^/ITGA6^high^ cells. E15.5 mouse hearts were flow sorted into ERBB-2^+^/ITGA6^high^ cells (EH) and plated on fibronectin-coated dishes. 10 s videos were recorded from spontaneously beating cells 24 h after plating.
(AVI)
