# Supplementary figures and images for: Differential Expression Levels of Integrin α6 Enable the Selective Identification and Isolation of Atrial and Ventricular Cardiomyocytes
Source: PLoS One. 2015 Nov 30;10(11):e0143538. doi: 10.1371/journal.pone.0143538 (PMC4664422; doi:10.1371/journal.pone.0143538)

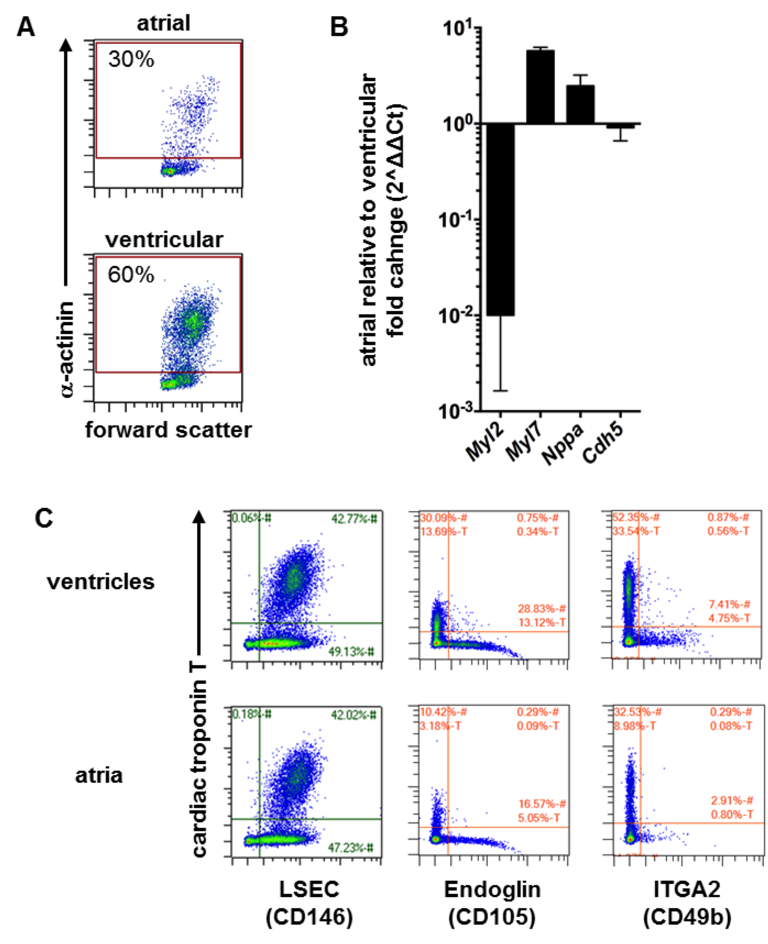

Supplement: S1 Fig — (A) Density plots show representative flow analysis of the cardiomyocyte content in both fractions as indicated by α-actinin staining. (B) Identity of atrial and ventricular cells was analyzed by qRT-PCR with regard to transcript levels of the ventricle-specific Myl2, encoding MLC-2v, the atrium-specific Myl7 (MLC-2a) and Nppa (ANF) as well as the endothelium-specific Cdh5 (VE-cadherin). Gene expression was calculated relative to Gapdh and analyzed using the ΔΔCT method (S1 Methods). The fold change is calculated as a relative change of atrial versus ventricular expression. Bar graph shows mean ± SD, n = 5. (C) Density plots, co-labeling of surface markers LSEC, Endoglin, and ITGA2 with intracellular cardiac troponin T. (TIF) [file pone.0143538.s001.tif]

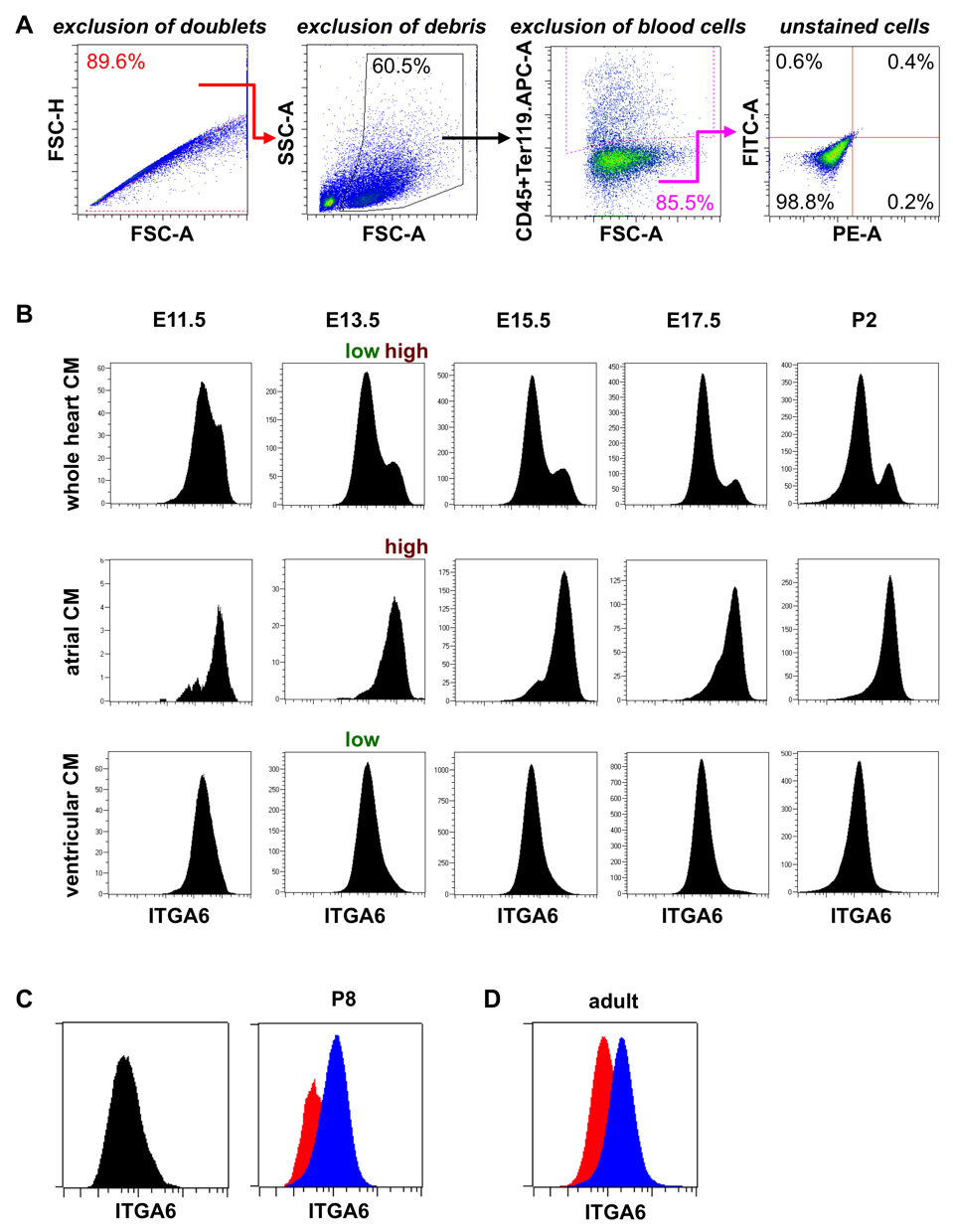

Supplement: S2 Fig — (A) Exemplary analysis. Determination of doublets by displaying FSC-A (X-axis) versus FSC-H (Y-axis); all events that are not on the diagonal are considered doublets and excluded from the analysis (NOT-gate, red dashed line). Definition of the cell population by displaying FSC-A versus SSC-A; only events of the main population are considered cells and included in the analysis (black line). Unless blood cells are not removed by lysis, they are labeled with antibodies against CD45 and Ter119 in one fluorescence channel (e.g. APC) and detected by displaying blood cells versus FSC-A; all CD45+ or Ter119+ cells are excluded from the analysis (NOT-gate, purple dashed line). Further analysis gates are set on unstained cells or secondary antibody controls. % refers to the entirety of the cells displayed in the plot which depend on the parent gates. (B) Representative flow analysis of mouse hearts of various developmental stages. Density plots, whole-heart cell suspensions were co-labeled with antibodies against ITGA6 and α-actinin. Histograms, ITGA6 expression gated on α-actinin+ cells of whole-heart (top row) and of mechanically separated atrial (mid row) and ventricular cells (bottom row). At all investigated stages atrial and ventricular CMs increasingly differ in ITGA6 expression intensity. (C) Histograms, cardiomyocytes were purified from P8 whole-heart, atria, and ventricles, and labeled with an antibody against ITGA6. There was only one peak detectable in the whole-heart preparation (left plot) but staining of isolated fractions revealed a remaining difference in ITGA6 expression intensity between the two fractions (right plot). Overlay: red peak = ventricular fraction, blue peak = atrial fraction. (D) Histogram, cardiomyocytes were isolated from adult atria and ventricles (S1 Methods), and were co-labeled with antibodies against ITGA6 and cardiac troponin T. There is a difference in ITGA6 expression intensity. Overlay: red peak = ventricular fraction, blue peak = at [file pone.0143538.s002.tif]

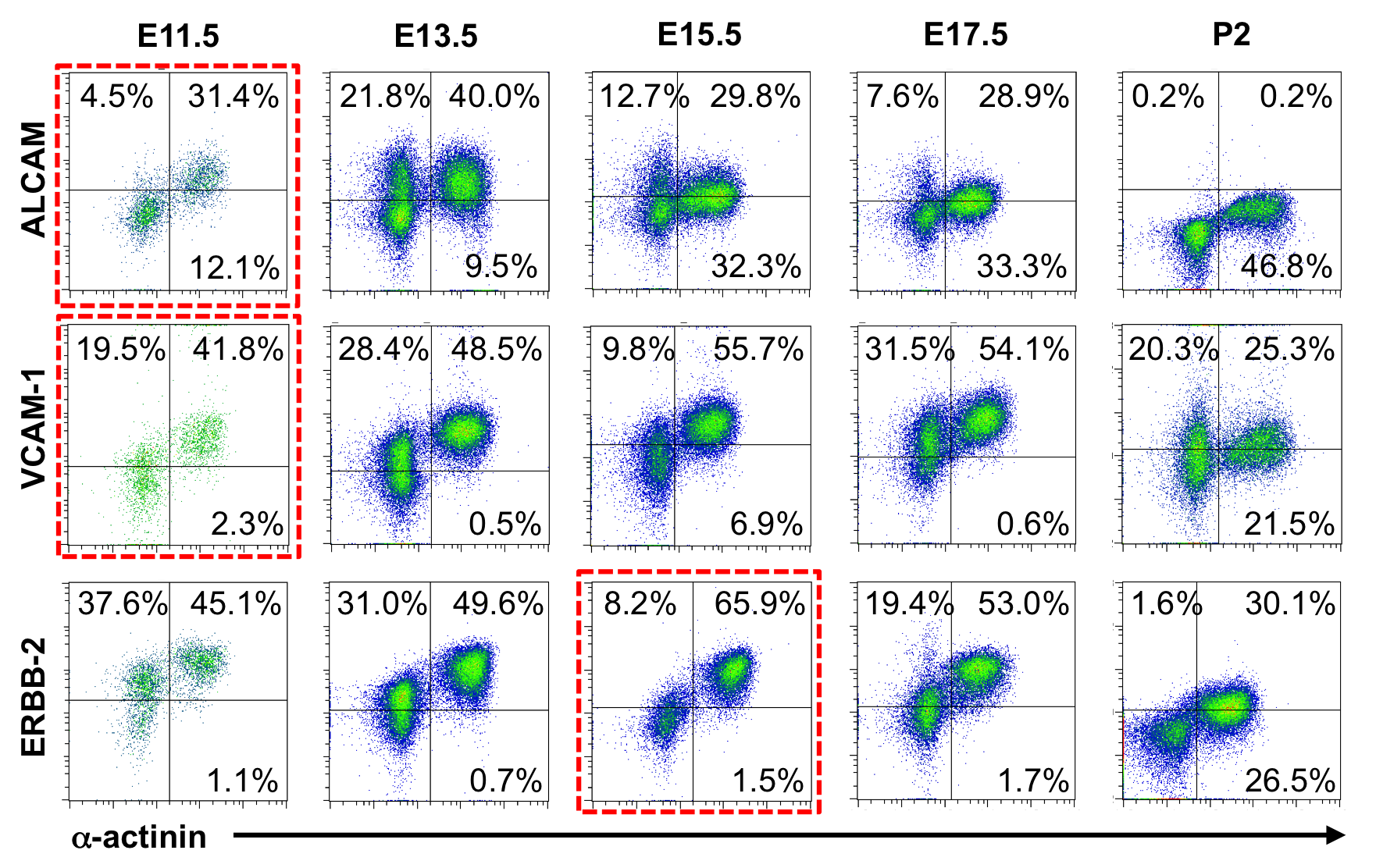

Supplement: S3 Fig — Representative flow analysis of mouse hearts of different developmental stages (E11.5 –P2). Density plots, whole-heart cell suspensions were co-labeled with antibodies against α-actinin (X-axis) and the surface markers ALCAM, VCAM-1 or ERBB-2 (Y-axis). Gates were set to corresponding unstained controls. As illustrated, cardiac expression of the markers was highly regulated during development. Cardiomyocyte-specific expression of ALCAM and VCAM-1 at E11.5 as well as of ERBB-2 at E15.5 is indicated by the red rectangle. (TIF) [file pone.0143538.s003.tif]

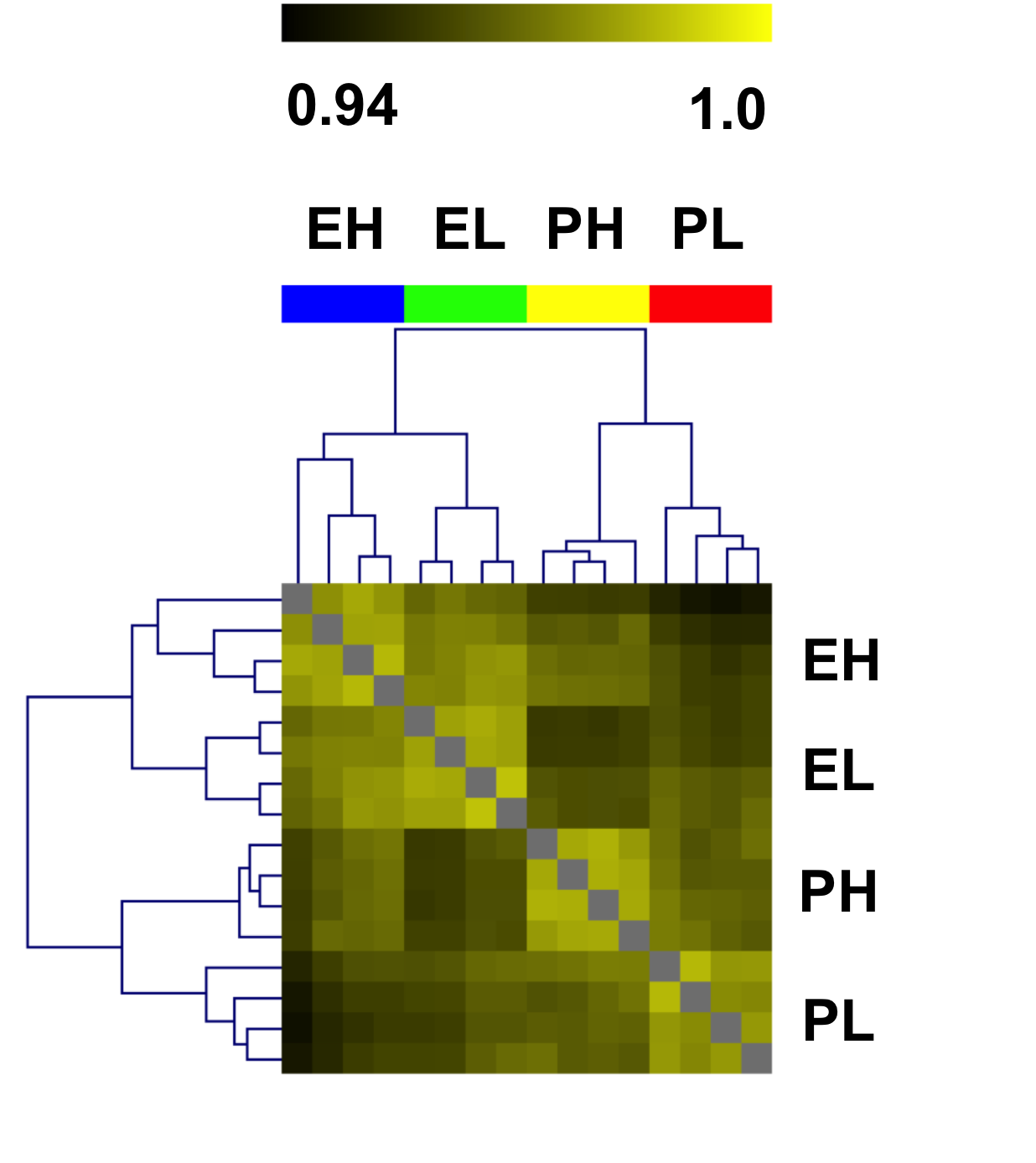

Supplement: S4 Fig — In four independent experiments embryonic and neonatal mouse heart cells were flow sorted and collected for gene expression analysis. The matrix was generated by unsupervised hierarchical clustering of pair-wise correlation coefficients (Pearson). Correlation coefficients are indicated by their color from 0.94 (black) to 1.0 (yellow). As depicted in the heat-map, hierarchical clustering of the complete dataset by experiments resulted in a clear separation of the different sample groups. EL = E15.5 ERBB-2+/ITGA6low, EH = E15.5 ERBB-2+/ITGA6high, PL = purified P2 CM ITGA6low, PH = purified P2 CM ITGA6high; n = 4. (TIF) [file pone.0143538.s004.tif]
