# Supplementary material for: Differential Expression Levels of Integrin α6 Enable the Selective Identification and Isolation of Atrial and Ventricular Cardiomyocytes
Source: PLoS One. 2015 Nov 30;10(11):e0143538. doi: 10.1371/journal.pone.0143538 (PMC4664422; doi:10.1371/journal.pone.0143538)
Supplement: S1 Methods — (DOC) [file pone.0143538.s005.doc]

**Supplemental Methods**

**Quantitative (q)RT-PCR analysis**

The transcript levels of *Myl2*, *Myl7*, *Nppa*, and *Cdh5* have been investigated by real-time quantitative PCR using PerkinElmer Applied Biosystems prism model 7000 sequence detection system (PE ABI 7000 SDS). RNA was isolated from dissociated atrial and ventricular fractions obtained from pools of 10 - 15 mouse embryos (CD1, wt, E13.5) using the NucleoSpin® RNA Kit (Macherey-Nagel). 6 ng of globally amplified cDNA libraries were used as template for each PCR analysis, all assays were performed in triplicates. Forward and reverse primer sequences were as follows: *Myl2*: CATTCAAGGTGTTTGATCCCG and TGCGAACATCTGGTCGATCTC, *Myl7*: AGGAGACCTATTCCCAGCTCG and CGAAGAGTGTGAGGAAGACGG, *Nppa*: AGAGACGGCAGTGCTCTAGGG and AGCGAGCAGAGCCCTCAGT, *Cdh5*: ATCTACGGCTACGAGGGCG and TCCCCAGTCGTTGAGGAAGT, *Gapdh*: ACCTGCCAAGTATGATGACATCA and TGCTGTTGAAGTCGCAGGAGACAA. The cycle conditions were: 95°C for 10 min followed by 40 repeats of 95 °C for 15 s and 60 °C for 1 min. For each amplified product, melting curves were determined according to the supplier’s guidelines ensuring specific amplification. For each run, negative controls were performed by omitting the template. The target gene expression was calculated relative to the *Gapdh* expression, analyzed using the ΔΔCT method and expressed as a relative fold change of atrial versus ventricular cell fraction.

**Dissociation of adult heart tissue**

Adult hearts (CD1, wt, 9 wks) were isolated and enzymatically dissociated using the Langendorff perfusion method as described in [S1] with minor variations. Animals were sacrificed by cervical dislocation. Hearts were dissected and perfused in a Langendorff aperture using oxygenated Ca^2+^-free Tyrode solution (135 mM NaCl, 4 mM KCl, 1 mM MgCl_2_, 2.5 mM HEPES, 5 mM Glucose, pH 7.4) with 25 mM 2,3-Butanedione monoxime (BDM) for 5 min. The enzymatic digestion with 1 mg/ml (activity 0.199 U/mg) Collagenase B and 0,033 mg/ml Trypsin was performed in oxygenated Tyrode solution supplemented with 25 mM BDM and 50 µM CaCl_2_ for 12 min. After enzymatic pre-incubation the atria were removed from the ventricles and further digested separately. Ventricles were manually dissected and the reaction was stopped in oxygenated Tyrode solution with 25 mM BDM, 50 µM CaCl_2_ and 5% FCS. The cell suspension was filtered through a 100 µm Cell strainer and centrifuged (700 rpm, 1 min).

**Flow cytometry analysis of surface marker expression kinetics during mouse heart development**

Mouse hearts were isolated from embryos of different developmental stages (E11.5 – E17.5) or from neonates (P2) and mechanically separated into atria and ventricles. Pooled whole hearts as well as atrial and ventricular fractions were dissociated into single cells manually (embryonic) or automated (neonatal). Up to 10^6^ single cells were incubated with antibodies in PBS containing 0.5 % BSA and 2 mM EDTA for 10 min at 4 °C. The antibodies used were: PE rat anti-mouse CD166 (ALCAM) (eBioscience, 1:100), Alexa Fluor® 647 rat anti-mouse CD106 (BD Pharmingen, 1:50), PE rat anti-mouse ErbB2/Her2 (R&D Systems, 1:10), for ITGA6 staining the PE rat anti-human and mouse CD49f antibody (Miltenyi Biotec, 1:11). In terms of blood cell exclusion embryonic tissue was co-labeled with PE or APC rat anti-mouse CD45 and rat anti-mouse Ter119 (both Miltenyi Biotec, 1:11). In order to co-label sarcomeric markers the Inside Stain Kit (Miltenyi Biotec) was used according to the manufacturer's instructions. Cells were incubated with 100 µL of Inside Fix (15 min, room temperature) and then incubated with the FITC-conjugated mouse anti-(sarcomeric) alpha actinin antibody (2 µg/mL) diluted in Inside Perm (10 min, rt). The labeled cells were resuspended in an adequate volume of staining buffer for flow cytometry analysis. Samples were measured on a MACSQuant® Analyzer (Miltenyi Biotec). Doublets, cell debris and eventually blood cells were excluded from the analysis (S2 Fig).

**Supplemental References**

S1. Stegemann M, Meyer R, Haas HG, Robert-Nicoud M. The cell surface of isolated cardiac myocytes - a light microscope study with use of fluorochrome-coupled lectins. J. Mol. Cell Cardiol. 1990;22:787-803.
